# *Cyclospora* spp. in Drills, Bioko Island, Equatorial Guinea

**DOI:** 10.3201/eid2003.131368

**Published:** 2014-03

**Authors:** Mark L. Eberhard, Jacob R. Owens, Henry S. Bishop, Marcos E. de Almeida, Alex J. da Silva

**Affiliations:** Centers for Disease Control and Prevention, Atlanta, Georgia, USA (M.L. Eberhard, H.S. Bishop, M.E. de Almeida, A.J. da Silva);; Drexel University, Philadelphia, Pennsylvania, USA (J.R. Owens, G. Hearn, S. Honarvar)

**Keywords:** Cyclospora spp. Cyclospora cayetanensis, parasites, drills, Mandrillus leucophaeus poensis, Bioko Island, Equatorial Guinea

**To the Editor:** More than a decade has passed since major outbreaks of *Cyclospora cayetanensis* infection in the United States and Canada drew attention to this newly emerging infection (*1**, **2*). Awareness of these infections was highlighted again by large outbreaks in the summer of 2013 (*3*). However, many questions remain unanswered regarding this organism, including aspects of its life cycle, geographic distribution, and range of related species.

In 1999, three new distinct *Cyclospora* species noted for their close similarity with *C. cayetanensis* from humans were isolated from monkeys in Ethiopia (*4*). A survey of primates in Kenya increased awareness of the extended distribution of these 3 species in eastern Africa and provided confirmation of their marked host specificity, even where the ranges of host species overlapped (*5*). Most recently, *C. colobi*–like organisms were identified in snub-nosed golden colobus monkeys in northwestern China (*6*). We report the characterization of *Cyclospora* spp. recovered from drills (*Mandrillus leucophaeus poensis*) on Bioko Island, Equatorial Guinea.

During January–February 2011 and 2012, fecal samples from free-ranging animals were collected and placed in 10% formalin (2011) or potassium dichromate (2012). Because samples were collected opportunistically from unidentified animals of undetermined age and sex, whether any samples were collected from the same animals in either year was not known. Fecal samples were concentrated by using the formyl ethyl acetate method, and sediment was examined by using fluorescent microscopy to detect oocysts (*4*).

Three (9%) of 26 samples from 2011 and 8 (31%) of 25 samples from 2012 were positive for *Cyclospora* oocysts that were spherical, measured 8–10 µm in diameter, and showed autofluorescence. The oocysts collected in potassium dichromate had sporulated by the time of examination, which facilitated and confirmed identification as *Cyclospora* spp. Representative samples from 4 animals in the second collection were submitted for molecular analysis.

The entire 18S rRNA gene (1,796 bp) was obtained from 2 DNA fragments amplified by PCR from DNA extracted from 3 fecal specimens by using procedures and primers for genetic analysis of coccidian parasites (*4*,*7*,*8*). Six distinct full-length 18S rRNA sequences were obtained and compared with sequences in GenBank.

Although our sequences showed high similarity with 18S rRNA genes for all *Cyclospora* species, the sequences were most similar to the *C. papionis* 18S rRNA gene (GenBank accession no. AF111187), even though 3 T→C transitions at nucleotides positions 680, 1054, and 1694 were observed. Analyses of these 6 sequences showed intravariation caused mainly by T→C and A→G transitions. Further studies on different species should be performed to verify whether this is a common feature in *Cyclospora* spp. 18S rRNA genes.

This report extends our knowledge of the range of *Cyclospora* spp. in monkeys to include western Africa and their host range to include an additional distinct primate species. Results of molecular analysis indicate that this *Cyclospora* sp. isolate from drills on Bioko Island is most similar to *C. papionis* from baboons in eastern Africa, an observation that is unexpected and somewhat difficult to explain. Previous studies have suggested that different primate hosts harbor distinctly different *Cyclospora* species (*4**–**6*). Baboons are not found on Bioko Island or in mainland Equatorial Guinea near Bioko Island and are allopatric with drills on the mainland. In addition, drills on Bioko Island have been separated from contact with drills on the mainland for 10,000–12,000 years (*9*), further isolating the ecology of this host–parasite relationship and confusing how *C. papionis* was established in drills on Bioko Island.

Drills are now considered to have closer phylogenetic affinity with mangabeys (*Cercocebus* spp.) than with baboons (*10*), although the phylogeny of these primates is not completely resolved. This finding further confuses an explanation of why the parasite isolated from drills would be similar to that recovered from baboons. It could be speculated that *C. papionis* arrived on Bioko Island from the mainland through some third host, such as collared (red-capped) mangabeys (*C. torquatus*), which has close phylogenetic relationships and overlapping ranges with drills and baboons. Any such explanation would mean that *Cyclospora* spp. infected drills before Bioko Island and the mainland separated.

Another possibility is that *Cyclospora* spp. exhibit host–niche specificity. Colobus monkeys, the host for *C. colobi*, are arboreal folivores, many of which consume relatively difficult-to-digest foods and have large specialized guts. Vervets, hosts for *C. cercopitheci*, are also arboreal, but have a frugivorous–insectivorous diet and consume little leaf matter. Baboons and drills, hosts for *C. papionis*, are predominantly terrestrial and have generalist–omnivorous diets and unspecialized guts. These different ecologic and physiologic differences among the 3 species may affect the observed *Cyclospora* spp. host specificity.

Observations in the present study extend our knowledge of the geographic and host range for cyclosporiasis. However, these observations leave several unanswered questions about our understanding of the parasite in nonhuman primates; the evolutionary relationship between human *C. cayetanensis* and these closely related species in monkeys; what additional monkey host species, especially on Bioko Island, may harbor *Cyclospora* spp.; and what other as yet unrecognized species of *Cyclospora* may be infecting primates.
